# L4 Pedicle Subtraction Osteotomy in a Patient With Multiple Previous Revisions: A Case Report

**DOI:** 10.7759/cureus.73534

**Published:** 2024-11-12

**Authors:** Alejandro J Quinonez, Austin H Carroll, Fred Mo

**Affiliations:** 1 Orthopedic Surgery, Georgetown University School of Medicine, Washington DC, USA; 2 Orthopedic Surgery, MedStar Georgetown University Hospital, Washington DC, USA

**Keywords:** neurosurgery, orthopedics, pedicle subtraction osteotomy, revision spine surgery, sagittal imbalance

## Abstract

Pedicle subtraction osteotomy (PSO) is a technically complex procedure that is effective at improving the sagittal profile in spinal deformity surgery. This case report describes a 64-year-old man with a history of ten previous spinal surgeries, including failed T10-pelvis posterior spinal fusion, undergoing revision with L4 PSO. The patient regained approximately 30° of lumbar lordosis. The procedure was complicated by an uneventful intraoperative durotomy and delayed postoperative surgical site infection requiring two surgical debridements and a prolonged course of antibiotics. At the 14-month follow-up, the patient was ambulating 3 miles per day and had significantly decreased pain with no sign of recurrent infection.

PSOs performed in revision cases are more challenging procedures but can achieve similar degrees of correction even in patients with multiple previous revisions.

## Introduction

Pedicle subtraction osteotomies (PSO) are complex procedures utilized to correct sagittal imbalance in spinal deformity surgery. PSOs increase the lordosis of the lumbar spine by creating a transpedicular wedge osteotomy in the targeted vertebral body to correct a pelvic incidence-lumbar lordosis (PI-LL) mismatch or elevated sagittal vertical axis (SVA). The sagittal balance represents the global stability of the spine required to keep the skull over the pelvis. This allows for a neutral gaze over the horizon with minimal energy utilization. Sagittal balance can be appreciated on a lateral full-length standing radiograph.

Excess anterior or posterior imbalance can predispose patients to worsening spinal deformity and symptomatology. Proper correction of the sagittal profile restores the “cone of economy” described by Hasegawa and Dubousset and thus decreases the energy expenditure required to maintain a patient’s horizontal gaze [1]. Previously published work has demonstrated the ability to gain approximately 30° of correction of lumbar lordosis following a PSO [2,3].

PI-LL, SVA, and pelvic tilt (PT) represent measurements of sagittal balance and have been shown to have a strong correlation with a patient’s health-related quality of life scores [4]. Sagittal and pelvic imbalances lead to compensatory mechanisms to maintain a horizontal gaze, which typically include retroversion of the pelvis and increased PT, which can lead to significant pain and decreased ambulatory ability [4].

In addition, the development of adjacent segment disease and proximal junction kyphosis has been associated with improper sagittal alignment [5-8]. Recent literature has focused on specifying various parameters to establish proper sagittal lumbar alignment and predict future segmental disease, including PI-LL, SVA, PT, cranial lordosis (L1-L4), caudal lordosis (L4-S1), and the lordosis distribution index [5]. PSOs, when conducted correctly to re-establish caudal lordosis, can, therefore, prevent further malalignment and adjacent disease in patients who have already had multiple surgeries.

PSOs are often technically complex and have high rates of morbidity and complications such as infection, neurologic injury, and incidental durotomy [9-11]. Complication rates in the literature have ranged from 15-24.3% in the setting of primary PSO [9,12,13]. Daubs et al. demonstrated in a retrospective review of 65 patients who underwent PSO with a major complication rate of 21.5% (neurologic deficit being the most common), a minor complication rate of 30.8%, and an intraoperative complication rate of 23%, with durotomy being the most common [14].

In the revision setting, when patients have had previous spinal surgery or fusion, PSOs become even more challenging due to distorted anatomy, diffuse scar tissue, and prior hardware that must be removed or revised. Each of these challenges can potentially increase the risk for any of the complications already seen in primary cases and potentially increase further with each subsequent revision. However, there is currently a paucity of literature describing PSOs performed in the setting of a revision, particularly in cases of patients who have had multiple failed revisions for sagittal deformity correction. In this report, we detail the case of a 64-year-old man with a history of 10 previous lumbar spine surgeries who underwent revision of his previous fusion and PSO at the L4 level.

## Case presentation

A 64-year-old male with a past history of 10 spinal surgeries, including T10-pelvis posterior spinal fusion, presented with chronic middle and lower back pain and complaints of prominences in his thoracic and sacral spine. At the time of presentation, his pain was significantly limiting his activity and ability to ambulate. He denied any changes in bowel or bladder function. On initial evaluation, the patient had 5/5 strength in his bilateral lower extremities, normal reflexes, and normal sensation bilaterally. He had notable prominences in the thoracic and sacral spine.

Initial diagnostic evaluation

**Figure 1:**
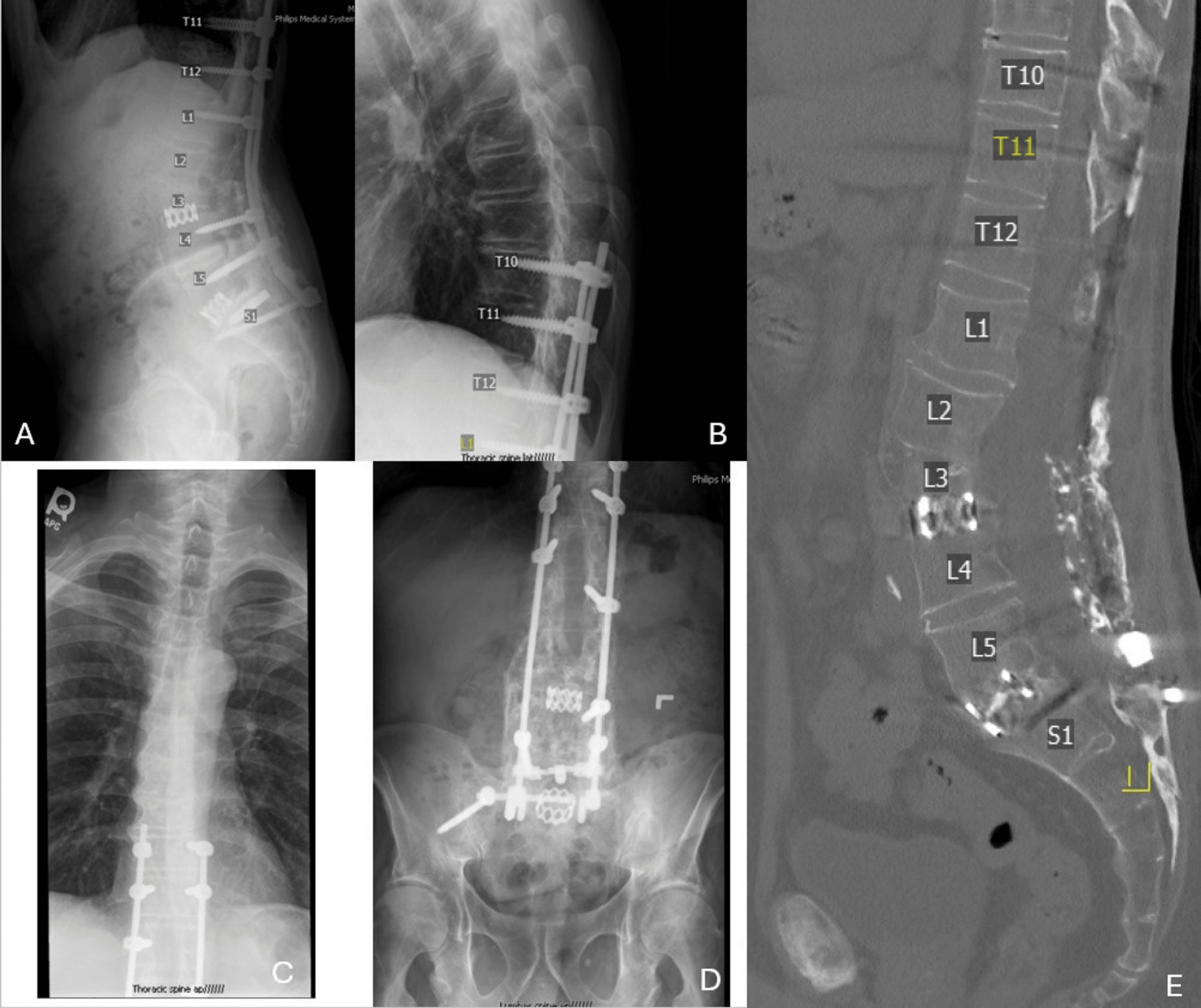
Preoperative lumbar images showing initial construct after 10 revisions A: preoperative lateral lumbar radiograph, B: preoperative lateral thoracic radiograph, C: preoperative AP thoracic radiograph, D: preoperative AP lumbar radiograph, E: sagittal lumbar CT AP: anteroposterior, CT: computed tomography

Initial imaging, including X-ray and computed tomography (CT) scans, demonstrated a previous fusion construct from T10-pelvis with interbody cages at L3-4 and L5-S1 (Figure 1). The S1 screws were noted to be broken bilaterally, and there was unilateral pelvic fixation on the right side. Fusion was seen throughout the construct; however, a fracture at the L3 vertebral body was noted. Unilateral screws on the left were present at L1 and L4. No screws were present at L2 or in the fractured vertebral body at L3. On MRI, there was no evidence of spinal stenosis.

Preoperatively, lumbar lordosis measured 34° with a PT of 31°, sacral slope of 34°, and pelvic incidence of 65°. There was, therefore, a PI-LL mismatch of 31°. Caudal lumbar lordosis from L4-S1 measured 21°.

Surgery

Due to the patient’s significant sagittal imbalance, failure of his previous hardware construct, and clinical symptoms, a revision was indicated with an L4 PSO to restore his sagittal balance. During the procedure, the prior fusion mass was identified and noted to have good bony fusion. The previous fusion mass was debrided, and a plane was developed to identify the dura. The previous set screws and rods were removed. Pedicle screws were inserted bilaterally at L2, and all pre-existing screws were checked for loosening and replaced if necessary. The previous screws in the sacrum were noted to be broken. Sacroiliac joint fixation and fusion were performed using an S2 sacral alar-iliac technique on the left side. Attention was then turned to the L4 PSO. The L3 and L4 nerve roots were identified and protected bilaterally. Both pedicles at L4 were carefully removed, and the lateral vertebral walls were dissected in a subperiosteal fashion with a retractor placed on either side. Temporary rods were utilized on either side as the osteotomy was performed under fluoroscopic evaluation. Set screws were then loosened, and under lateral fluoroscopy, the osteotomy was gradually closed. Temporary rods were then replaced with permanent rods. Rod-to-rod connectors were placed along with two additional rods on each side of the PSO to achieve a quad-rod construct. A dural tear was then noted at the L3 nerve root, which was repaired with a glue patch repair. A Valsalva maneuver was performed, and no further leak could be identified. The wound was sealed and irrigated with normal saline. Throughout the case, somatosensory evoked potentials, and EMG remained stable. Blood loss was estimated to be 2.4 liters, and the patient received two units of blood. The plastic surgery service was consulted for assistance with paraspinal flap closure. Operative time was 216 minutes.

Postoperative course

**Figure 2:**
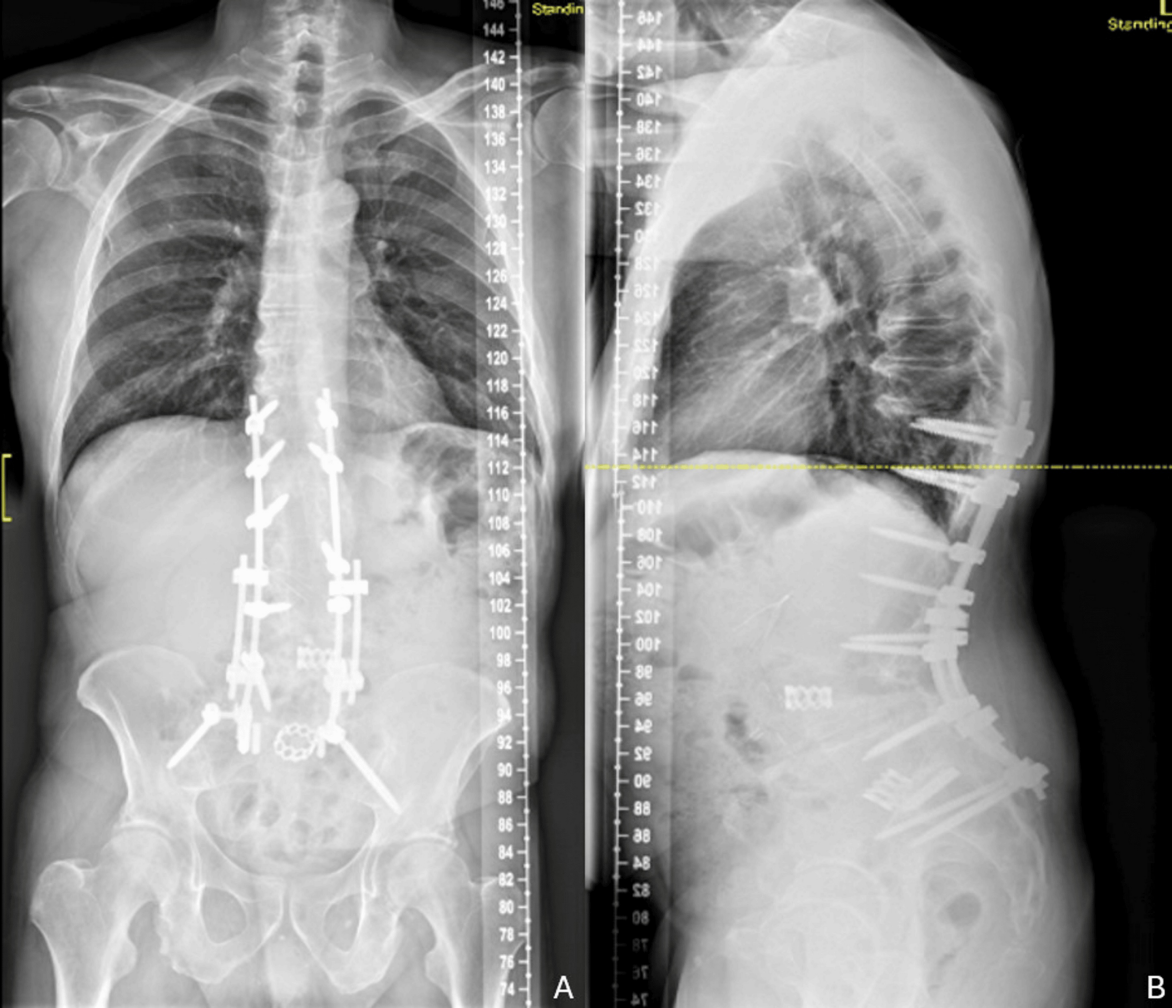
Postoperative lumbar images showing new construct after L4 PSO and revision posterior spinal fusion A: postoperative AP radiograph, B: postoperative lateral radiograph AP: anteroposterior, PSO: pedicle subtraction osteotomy

On postoperative imaging, lumbar lordosis increased to 61° and PT decreased to 19°. Caudal lumbar lordosis (L4-S1) measured 34.1° (Figure 2). Postoperatively, the patient experienced anemia that resolved with a blood transfusion on postoperative day three and another on postoperative day four. The remainder of the patient’s postoperative hospital stay was uncomplicated.

At the three-month postoperative visit, the patient’s incision was well healed, and he reported being able to ambulate approximately 1.5 miles per day with significant improvement in his pain. He continued to do well until approximately 11 months postoperatively, at which point he received an epidural injection with pain management. Shortly after, he developed purulent drainage from his incision, and a CT scan was obtained, which demonstrated a paraspinal abscess. He was admitted to the hospital and underwent two debridements followed by closure with the plastic surgery service. Intraoperative cultures were positive for proteus mirabilis and the infectious disease service was consulted. He was placed on two months of intravenous ceftriaxone, followed by a 12-month course of amoxicillin/clavulanic acid. At the last follow-up, 14 months postoperatively, his incision was well healed, and he was ambulating 2-3 miles per day with no sign of recurrent infection.

## Discussion

Severe sagittal imbalances in patients with prior failed fusion constructs remain a challenge for the treating spine surgeon. In this case report, our patient with 10 previous revision spinal surgeries and failed T10-pelvis posterior spinal fusion was able to achieve an increase of approximately 30° in their lumbar lordosis after an L4 PSO through their previous fusion mass.

In recent literature, there has been increased focus on the proper understanding of achieving properly distributed lumbar lordosis across all segments of the lumbar spine. Pesenti et al. demonstrated that the L4-S1 segments contribute approximately 60% of the global lumbar lordosis. In cases where <50% of the global lumbar lordosis was attributed to the L4-S1 segments, there were increased rates of adjacent segment disease requiring further revision [6,7]. PSO can thus help achieve proper global and segmental lordosis to further decrease the risk of adjacent segment disease and the need for future revision.

The correction achieved in this patient is similar to that of other reports with primary PSO [2,3]. In their study, Gupta et al. found a higher rate of major complications in the revision group (31.9%) compared to PSO in primary surgery (24.3%); however, this was not deemed statistically significant (p=0.207) [9]. In this study, the highest reported major complication in both groups was increased intraoperative bleeding greater than 4 liters [9]. In our case, blood loss was 2.4 liters with an operative time of three hours and 36 minutes.

Patel et al. reported on a series of patients with PSO correction for flatback following previous spinal surgery [13]. Three of the 17 (17.6%) patients in their study had a complication, which included infection in two patients and cauda equina syndrome in one patient [13]. In their series, Yahanda et al. reported a postoperative complication rate of 15% encompassing rod breakage, proximal junctional kyphosis, and wound infection [12]. Another series by Otani et al., which reviewed the cases of PSO in revision surgery, reported an intraoperative rate of 29.5% with neurologic deficit as the most common complication [15]. Many of these reviews detail reports of cases of revision after a single initial procedure, and further review of cases that involve patients with a history of multiple revisions is needed. PSOs can be completed in revision settings, but additional care and attention should particularly be given to loss of blood and neuromonitoring throughout the procedure. Further, there is a significant risk of infection from a PSO, which is further amplified in the multiply-operated patient in revision settings.

## Conclusions

PSO remains an effective solution for restoring lumbar lordosis in cases of sagittal imbalance, even in patients who have had multiple previous lumbar spine surgeries. In the revision setting, PSO is a technically difficult procedure that is frequently complicated by unintentional durotomy, significant blood loss, infection, and neurologic injury. The literature would benefit from additional work describing PSO outcomes in patients who have had multiple failed previous spinal surgeries.
